# Sports Nutrition for Young, Older, and Female Athletes; Plant-Based Ingredients; and Return to Play During COVID-19

**DOI:** 10.1007/s40279-021-01517-7

**Published:** 2021-09-13

**Authors:** Lawrence L. Spriet

**Affiliations:** Human Health and Nutritional Sciences, University of Guelph, Guelph, ON N1G 2W1 Canada

This supplement examines the latest sports nutrition information relating to youth, older, and female athletes. It also examines where the scientific field stands on the role of plant-based protein for athletes and the controversial area of cannabis and cannabidiol in athletic performance and recovery. Lastly, the supplement explores the views of sports practitioners as they dealt with the early months of the COVID-19 pandemic and the issues surrounding “return to play” for athletes in all sports. The past 18 months have been a sobering awakening to the dangers of an uncontrolled virus and the suffering and loss of life that has occurred throughout the world. We are now emerging from this pandemic with the help of vaccines and physical distancing measures. Many people found solace and relief through exercise during the pandemic. In addition, the loss of organized sport at all levels made it very clear that exercise and sport play important roles in the lives of many people. We hope that the papers in this supplement will help re-focus people on the importance of nutrition for sustaining healthy exercise and sport for people of all ages and skill levels.

The Gatorade Sports Science Institute (GSSI) has been bringing sports nutrition and sports science researchers together for ~ 36 years to discuss many topics relating to the performance and well-being of athletes. Since 2012, these meetings have been known as the GSSI Expert Panel. The worldwide COVID-19 pandemic necessitated that the latest meeting, in October 2020, was held in a virtual format. Despite this, the meeting was a great success, and the authors subsequently summarized the recent work in their topic area, resulting in the manuscripts in this *Sports Medicine* supplement (the eighth in a series supported by GSSI).

The first paper in this supplement examines the importance of nutrition during the years of adolescence (13–18 years), which is a period of growth and physical development with changes in body composition, metabolic and hormonal fluctuations, maturation of organ systems, and establishment of nutrient deposits [1]. Adequate energy is needed for both growth and athletic activities in young athletes. To maximize their potential, adolescent athletes need to adopt eating patterns that prioritize sound physical and mental development and sound sport nutrition guidelines [1].

The next two papers examine nutritional questions related to master athletes aged ≥ 60 years [2, 3]. Moore [2] argues that, although there is some skeletal muscle anabolic resistance to the ingestion of protein with aging, master athletes generally have muscle characteristics, physiological responses to exercise, and protein metabolism similar to those of young athletes. Consequently, their protein requirements do not appear vastly different from those of young athletes, although more research is needed in the master athlete population. As we age, our performance decreases, and considerable information exists to suggest that long-chain polyunsaturated n-3 fatty acid ingestion may promote skeletal muscle anabolism and strength in untrained older people and improve markers of muscle recovery following damaging exercise in younger people [3]. Research is needed to determine whether polyunsaturated n-3 fatty acid ingestion can promote and sustain muscle growth and strength, and recovery from exercise, in trained master athletes. Ingestion of n-3 fatty acid supplementation may lead to improvements via alteration of the lipid profile of the sarcolemmal, mitochondrial, and sarcoplasmic reticulum membranes in skeletal muscle [3].

It is abundantly clear to everyone working in science that females and female athletes have been understudied. The fourth paper in this supplement addresses the latest recommendations for fueling female athletes given the differences in substrate metabolism between women and men [4]. The paper also discusses the menstrual patterns of female athletes, nutrient and hydration needs during different menstrual cycle phases, and health and performance issues related to menstrual cycle disruption. Importantly, areas for future research are identified, and important methodological considerations for the required investigations are discussed [4].

The fifth paper in this supplement addresses the global trend of a transition towards the consumption of a more plant-based diet, which includes an increase in the consumption of plant-based proteins over animal-based proteins [5]. This way of eating is also very popular in sports nutrition, and it is known that ingesting proteins derived from soy and wheat results in lower post-meal muscle protein synthesis rates than does ingesting an equivalent amount of animal-derived protein. This raises the concern that athletes ingesting mainly plant-based protein may need to ingest more protein [5]. However, the authors discuss ways that athletes can compensate for the lower anabolic effect of plant-based proteins and that additional research comparing plant- and animal-based proteins in foods is warranted.

The sixth paper examines the plant product cannabis, which is widely used for both recreational and medicinal purposes [6]. Although cannabis use is illegal in many countries, a growing number of countries have legalized its use. In the sports world, there is interest in the potential use of cannabis and its constituents for athletic performance and recovery. However, the effects of the two most abundant cannabis constituents, delta-9-tetrahydrocannabinol (THC) and cannabidiol (CBD), have been largely uninvestigated in the sports world. Clearly, the potential of THC to affect performance has not been properly studied, but there is some evidence that CBD may improve sleep quality, pain, and mild traumatic brain injury in certain athletes [6]. The next few years will see an increase in research examining the effects of THC and CBD on performance and recovery from exercise in athletic populations.

The final paper, by Ibiebele and Chiampas [7], details the practitioner’s perspective in dealing with the first few months of the COVID-19 pandemic, and the planning involved in returning athletes to play. Fortunately, the plan needed to respond to the pandemic was already in place as previous events established the importance of developing “incident command structures” to streamline communication, maximize coordination, and establish contingencies for each new emergency. The responses to the pandemic included appropriate hygiene and physical distancing, use of masks, rigorous monitoring and screening of symptoms, widespread testing, comprehensive contact tracing, and considerations for travel and facilities [7].

The papers in this supplement have collectively identified many areas of sports nutrition that require additional research so that all members of the athletic population are adequately advised. We hope the readers of these papers are inspired to generate new knowledge and disseminate the present knowledge related to these topics.
